# Co-quantification of crAssphage increases confidence in wastewater-based epidemiology for SARS-CoV-2 in low prevalence areas

**DOI:** 10.1016/j.wroa.2021.100100

**Published:** 2021-04-06

**Authors:** Maxwell L. Wilder, Frank Middleton, David A. Larsen, Qian Du, Ariana Fenty, Teng Zeng, Tabassum Insaf, Pruthvi Kilaru, Mary Collins, Brittany Kmush, Hyatt C. Green

**Affiliations:** a Department of Environmental and Forest Biology, SUNY-ESF, Syracuse, NY 13210; b Department of Neuroscience and Physiology, Upstate Medical University, Syracuse, NY 13210; c Department of Public Health, Syracuse University, Syracuse, NY 13244; d Quadrant Biosciences, Syracuse, NY 13210; e Department of Civil & Environmental Engineering, Syracuse University, Syracuse, NY 13244; f Bureau of Environmental and Occupational Epidemiology, New York State Department of Health, Albany, NY 12337; g Department of Epidemiology and Biostatistics, University at Albany, Rensselaer, NY 12144; h Department of Environmental Studies, SUNY-ESF, Syracuse, NY 13210

**Keywords:** Wastewater-based epidemiology, Viral concentration, Cross-assembly phage, SARS-CoV-2

## Abstract

Wastewater surveillance of SARS-CoV-2 RNA is increasingly being incorporated into public health efforts to respond to the COVID-19 pandemic. In order to obtain the maximum benefit from these efforts, approaches to wastewater monitoring need to be rapid, sensitive, and relatable to relevant epidemiological parameters. In this study, we present an ultracentrifugation-based method for the concentration of SARS-CoV-2 wastewater RNA and use crAssphage, a bacteriophage specific to the human gut, to help account for RNA loss during transit in the wastewater system and sample processing. With these methods, we were able to detect, and sometimes quantify, SARS-CoV-2 RNA from 20 mL wastewater samples within as little as 4.5 hours. Using known concentrations of bovine coronavirus RNA and deactivated SARS-CoV-2, we estimate recovery rates of approximately 7-12% of viral RNA using our method. Results from 24 sewersheds across Upstate New York during the spring and summer of 2020 suggested that stronger signals of SARS-CoV-2 RNA from wastewater may be indicative of greater COVID-19 incidence in the represented service area approximately one week in advance. SARS-CoV-2 wastewater RNA was quantifiable in some service areas with daily positives tests of less than 1 per 10,000 people or when weekly positive test rates within a sewershed were as low as 1.7%. crAssphage DNA concentrations were significantly lower during periods of high flow in almost all areas studied. After accounting for flow rate and population served, crAssphage levels per capita were estimated to be about 1.35 × 10^11^ and 2.42 × 10^8^ genome copies per day for DNA and RNA, respectively. A negative relationship between per capita crAssphage RNA and service area size was also observed likely reflecting degradation of RNA over long transit times. Our results reinforce the potential for wastewater surveillance to be used as a tool to supplement understanding of infectious disease transmission obtained by traditional testing and highlight the potential for crAssphage co-detection to improve interpretations of wastewater surveillance data.

## Highlights

• Ultracentrifugation with a sucrose cushion allowed sensitive detection of SARS-CoV-2 wastewater RNA.
• Per capita levels of crAssphage were consistent across sites supporting its use as a surrogate for SARS-CoV-2.
• Limits of quantification corresponded to daily positive tests as low as 0.37 per 10,000 people, depending on the area.
• SARS-CoV-2 RNA signal strength is associated with increased COVID-19 cases in the following 7 days.
• SARS-CoV-2:crAssphage ratios were significantly associated with COVID-19 cases.

## Graphical abstract

**Figure:**
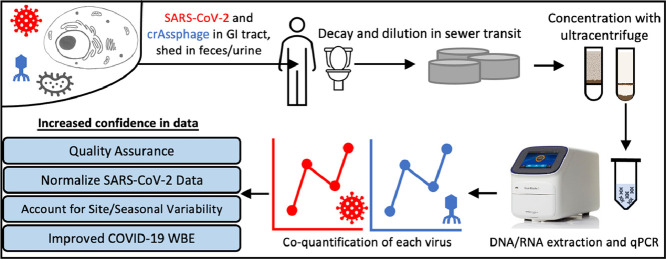

## 1 Introduction

Severe acute respiratory syndrome coronavirus 2 (SARS-CoV-2) has infected over 130 million people and has been attributed to over 2.8 million deaths globally as of April 9^th^, 2021 (WHO, 2021). While the primary mechanism of transmission for SARS-CoV-2 is through respiratory droplets and aerosols (Meselson, 2020), viral RNA has also been detected in the gastrointestinal system, feces, and urine of infected persons (Chen et al., 2020; Y. Wu et al., 2020; Singer and Wray, 2020). Notably, viral RNA levels in the sputum and stool of patients with mild COVID-19 symptoms are similar to that found in the upper respiratory tract (Wölfel et al., 2020). The presence of a substantial quantity of viral RNA in feces and urine provides the opportunity for wastewater-based epidemiology (WBE) approaches to be applied to the surveillance of COVID-19, tracking the emergence of disease and transmission trends over time (Daughton, 2020; Farkas et al., 2020). Such monitoring efforts are currently being explored worldwide in an attempt to bolster the public health response to the pandemic. A robust and effective wastewater monitoring program for SARS-CoV-2 could help to inform resource allocation decisions (e.g. where to prioritize testing and contact tracing), target community interventions such as social distancing measures or other restrictions, and provide an additional tool by which policy makers could assess when and how to reopen local economies (Mallapaty, 2020, McMinn et al., 2021; Larsen et al., 2020; Daughton, 2020). Additionally, an effective wastewater monitoring approach could be used in the surveillance of facilities such as jails, university dormitories (Colosi et al., 2020), and assisted living facilities which may be especially susceptible to COVID-19 outbreaks, providing officials with the tools to limit the spread of the virus both within and from these types of facilities.

Numerous groups have reported methods for the detection of SARS-CoV-2 in wastewater following global outbreaks of COVID-19 in early 2020. These approaches to concentrate viral particles and RNA have included combinations of low-speed centrifugation and centrifugal filters (Medema et al., 2020; Ahmed et al., 2020a; Nemudryi et al., 2020b; Alpaslan Kocamemi et al., 2020), polyethylene glycol (PEG)-precipitation (Zhang et al., 2020; Alpaslan Kocamemi et al., 2020; F. Wu et al., 2020b; La Rosa et al., 2020; Colosi et al., 2020), aluminum-driven flocculation (Randazzo et al., 2020), filtration through charged membranes (Ahmed et al., 2020a; Colosi et al., 2020), and ultracentrifugation (Wurtzer et al., 2020; Zhang et al., 2020; Ampuero et al., 2020; Colosi et al., 2020). While many of these methods can be applied to wastewater surveillance with varying degrees of success (as reviewed in Kitajima et al., 2020; Ahmed et al., 2020b; Rusiñol et al., 2020; Colosi et al., 2020), their practical application into large scale monitoring efforts can be limited by factors such as turnaround time (i.e. the time from sample acquisition to data generation) and dependence on supply chain continuity for single use materials such as charged membranes or centrifugal filtration units. Ultracentrifugation is an attractive approach because once the initial investment in equipment is made, the material cost and sample processing time are low. Furthermore, the ability to manipulate the amount and viscosity of the sedimentation medium as well as the ultracentrifugation time and speed permit partial nucleic acid purification concurrent with concentration.

While several studies have found correlations between viral concentrations in wastewater and the number of confirmed COVID-19 cases (Nemudryi et al., 2020a, NYS GIS - Parcels [WWW Document] n.d, NYS Street Address Mapping (SAM) [WWW Document] n.d; Bar Or et al., 2020, Barril et al., 2021; Ampuero et al., 2020), attempts at quantifying the numbers of infected individuals have varied in their success (Medema et al., 2020; Ahmed et al., 2020a; F. Wu et al., 2020a; Vallejo et al., 2020). Several studies have also indicated that wastewater monitoring of SARS-CoV-2 could provide an early warning sign for viral outbreaks in the community, with viral RNA being detected in wastewater samples prior to positive clinical testing of individuals (Medema et al., 2020; La Rosa et al., 2020; Randazzo et al., 2020). With either approach, some measure of the effects of decay and dilution in the wastewater infrastructure is needed so that changes in SARS-CoV-2 RNA over time or between sampling locations better reflect changes in infection levels versus changes in weather, water usage, or other factors. Co-quantification of viruses that are abundant in the human gut, such as pepper mild mottle virus (PMMoV) (D'Aoust et al., 2021a, D'Aoust et al., 2021b, Daughton, 2020; F. Wu et al., 2020b), to act as surrogates for SARS-CoV-2 is the prevailing approach although it is not completely clear which surrogates mimic the behavior of SARS-CoV-2 in the wastewater system most closely. Furthermore, for surrogates with a DNA genome, expressed RNA can simultaneously be measured, which may more closely reflect the decay of SARS-CoV-2 RNA in wastewater compared to DNA targets. Non-viral surrogates such as nicotine, cortisol, and creatinine have also been proposed as normalizers in WBE applications, although the quantification of these targets may be challenging and variability in consumption and disposal rates can create uncertainties in analysis (Polo et al., 2020; Rico et al., 2017).

In this study, our goals were to a) develop a reliable and scalable method for detecting and quantifying SARS-CoV-2 wastewater RNA from areas with low infection rates and b) integrate co-quantification of viral nucleic acids from crAssphage, an abundant human gut bacteriophage, into SARS-CoV-2 wastewater monitoring to help account for sources of both inter- and intra-sewershed variability. We measured both crAssphage DNA and RNA because it is currently unclear which serves as a better fecal normalizer when trying to associate SARS-CoV-2 wastewater RNA concentrations to relevant epidemiological parameters. Ultracentrifugation optimization trials were initially conducted prior to analyzing 181 wastewater influent samples collected from six Upstate New York counties.

## 2 Materials and methods

### 2.1 Sampling locations and sample collection and transport

Twenty-four-hour composite influent wastewater samples (110 mL – 1.9 L) were collected from 28 different access points in combined sewage networks across Upstate New York in Onondaga, Cayuga, Cortland, Tompkins, Oswego, and Warren Counties (Table 1, Figure S1). Information on the age of wastewater in these systems was only available for six Onondaga County access points (Table S1), where mean transit time ranged from 1.2 to 4.4 hours (Wang et al. 2020). Samples were stored at approximately 4 °C following collection and were transported on ice to Upstate Medical University (Syracuse, NY) the following morning for processing and viral concentration (with the exception of Onondaga County samples collected on the 28^th^ of April, which were frozen at -20 °C for processing at a later date following methodological optimizations). From April 28^th^ to June 24^th^, 2020 a total of 181 wastewater samples were collected and processed for the detection of SARS-CoV-2 and crAssphage nucleic acids. During the sample collection process, influent flow rate, pH, and water temperature were also measured at some access points. Average daily minimum air temperature in each county during this time period ranged from 9.2 to 14.6 °C, average daily maximum air temperature ranged from 21.8 to 25.9 °C, average daily precipitation ranged from 0.07 to 0.21 cm, and daily relative humidity ranged from 36 to 86% (NOAA). Information on the topographical area of each sewershed was accessed through New York State and/or County databases and the size of the population served was estimated using census data. Characteristics of individual access points within each county are summarized in Table S1.

**Table 1:** Summary of locations and dates of sampling in New York State

**County**	**Appx. Population**	**Average Daily Incidence**^a^	**Test Positivity**^b^**(%)**	**N Access Points Sampled**	**Time Period Sampled**	**Total N Samples**
Onondaga	460,000	34.5	2.94	16	4/28 – 6/24/2020	122
Cayuga	75,000	1.1	0.47	4	5/19 – 6/22/2020	24
Warren	70,000	0.4	0.20	3	5/27 – 6/23/2020	15
Oswego	120,000	3.6	1.10	2	6/03 – 6/23/2020	8
Tompkins	100,000	0.5	0.16	2	6/02 – 6/22/2020	7
Cortland	50,000	0.2	0.11	1	5/27 – 6/22/2020	5

a Average daily incidence is calculated as the total number of new positive cases that occurred over the time period sampled divided by the length of the time period (days).

b Test positivity is calculated as the total number of positive tests out of the total number of tests performed in each county during the time period sampled. Diagnostic test results include the results of both PCR and antigen tests.

### 2.2 Ultracentrifugation of wastewater through a sucrose cushion

**Fig. 1:**
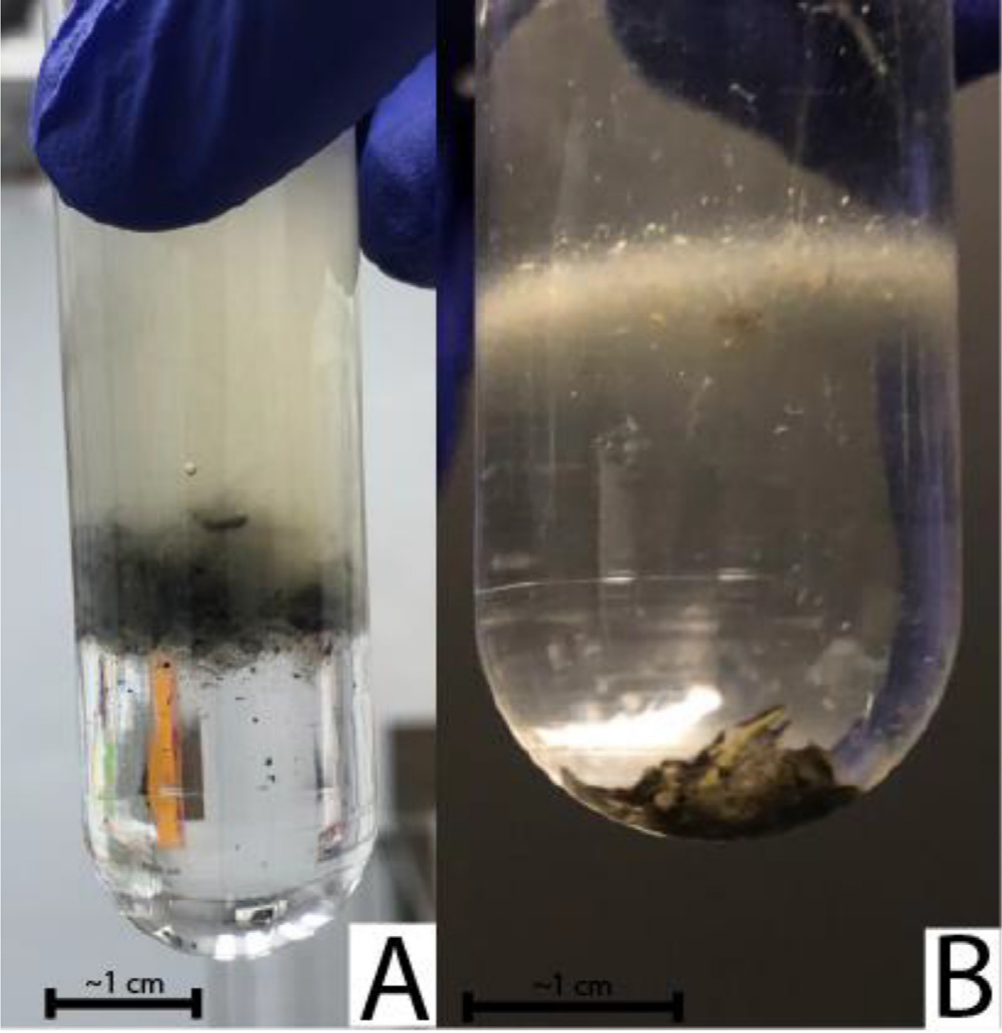
(A) Raw influent wastewater above 50% sucrose solution prior to ultracentrifugation. (B) Pellet produced by 45 minutes of ultracentrifugation and residual debris on top of the sucrose cushion.

Prior to ultracentrifugation, wastewater samples were blended to resuspend particulates that had settled during transport or storage. Twenty milliliters were transferred into a disposable 38.5 mL ultracentrifuge tube (Product No. 75000471, ThermoFisher®, Mass., USA) using a disposable serological pipette. Unless otherwise noted in the optimization experiments described in the section 2.3, a 12 mL sucrose cushion (50% sucrose in TNE buffer [20 mM Tris-HCL (pH 7.0), 100 mM NaCl, 2 mM EDTA]) was then carefully added underneath the wastewater using a serological pipette so that wastewater and the sucrose solution formed distinct layers in the ultracentrifuge tube (Fig. 1A). In batches of six, samples were balanced by the addition of distilled water (<500 μL) and then ultracentrifuged at 150,000 × g at 4 °C on a Sorvall® WX Ultra series with a Sorvall® SureSpin® 630 (6 ×36 mL) Swinging-Bucket Rotor (ThermoFisher®). Prepared samples were ultracentrifuged for 45 minutes unless otherwise noted for optimization experiments. Following ultracentrifugation and the generation of pellets containing viral particles and nucleic acids (Fig. 1B), the supernatant was carefully decanted with a new serological pipette and pellets were resuspended in 200 μL 1X PBS and transferred to 1.7 mL microcentrifuge tubes. Resuspended pellets were stored at -20 °C for <24 hours until nucleic acid extraction. Replicates were processed for optimization experiments only.

### 2.3 Optimization of viral nucleic acid recovery

Samples collected from Onondaga County on April 28^th^, May 6^th^, and May 13^th^, 2020 were used to perform optimization experiments. To identify sucrose concentrations and ultracentrifugation times that resulted in higher levels of viral nucleic acid recovery, we used crAssphage as a surrogate since native SARS-CoV-2 concentrations were too low to be used as reliable indicator of recovery. First, well-blended wastewater subsamples were ultracentrifuged with 20, 50, and 70% sucrose cushions for 20, 90, and 120 minutes, respectively, with lower concentration cushions receiving shorter ultracentrifugation times. Pellets were then analyzed for crAssphage DNA.

Next, once the optimal sucrose concentration was identified, we tested the effect of reducing ultracentrifugation time by making six replicate subsamples and ultracentrifuging for 30 (n=2), 45 (n=2), and 75 minutes (n=2) while holding sucrose concentration constant. Pellets were then analyzed for both crAssphage DNA and RNA.

Then, to estimate the efficiency with which SARS-CoV-2 RNA is pelleted, 20 mL wastewater aliquots (n =2) with low initial concentrations of SARS-CoV-2 RNA (appx. 8 genome copies per mL) were spiked (spike equivalent to appx. 580 genome copies per mL wastewater) with heat deactivated SARS-CoV-2 (Catalog No. NR-52286, BEI Resources®, Virginia, USA) prior to ultracentrifugation for 45 minutes. After ultracentrifugation, the following layers were analyzed: aqueous upper (top 10 mL), aqueous lower (second 9 mL), cushion interface (1.5 mL, targeting particles suspended just above the sucrose cushion), sucrose upper (6 mL), sucrose lower (6 mL), and pellet (appx. 200 uL). Two hundred microliter subsamples of each layer and the resuspended pellets were then analyzed for recovery of SARS-CoV-2 RNA, crAssphage DNA, and crAssphage RNA.

Finally, to estimate the loss of nucleic acids through the nucleic acid purification procedure specifically, wastewater pellets (n = 7) generated from homogenized samples originating from several access points were spiked with appx. 125,000 genome copies of bovine coronavirus (BCoV) RNA extracted from a vaccine (Bovine Rotavirus-Coronavirus Vaccine from Zoetis, NJ, USA). Extraction of total nucleic acids from wastewater pellets was carried out by the method described below. BCoV RNA concentrations were determined via RT-qPCR using a previously published assay (Decaro et al., 2008, Table S2). In routine processing, deactivated SARS-COV-2 and bovine coronavirus vaccine were not used to estimate nucleic acid recoveries. Instead, crAssphage was used to confirm recovery of nucleic acids and as a fecal normalizer for SARS-COV-2.

### 2.4 Nucleic acid extraction and synthesis of crAssphage cDNA

Total nucleic acids were extracted from resuspended pellets using the AllPrep® PowerViral® DNA/RNA Kit (Qiagen®, Hilden, Germany) according to manufacturer's protocol with the omission of the optional bead beating step. Nucleic acids were eluted in 50 μL elution buffer, five of which was used immediately to generate total cDNA using the QuantiTect® Reverse Transcription Kit (Qiagen®) according to manufacturer's protocol to allow estimation of crAssphage RNA. Total nucleic acid samples and cDNA were immediately stored at -80 °C until viral quantification via RT-qPCR and qPCR.

### 2.5 Quantification of viral nucleic acids

RT-qPCR was used to detect the presence of SARS-CoV-2 RNA in undiluted total nucleic acid extracts using a multiplex reaction with the previously published IP2 and IP4 assays targeting separate regions of the RdRp gene (Institut Pasteur, 2020). Reactions consisted of 6.25 μL Reliance One-Step Multiplex RT-qPCR Supermix (Bio-Rad®, California, USA), 0.4 μM each primer, 160 nM each probe (both HEX), molecular grade water, and 2.5 μL nucleic acid template for a total reaction volume of 25 μL. Thermal cycling conditions were 10 minutes at 50 °C, 10 minutes at 95 °C, followed by 45 cycles of 95 °C for 10 seconds and 59 °C for 30 seconds. crAssphage nucleic acids were quantified using the previously published CPQ_056 assay (Stachler et al., 2017). Reactions consisted of 12.5 μL TaqMan® Environmental MasterMix (ThermoFisher®), 1 μM primers, 80 nM probe, molecular grade water, and 2 μL nucleic acid template for a total reaction volume of 25 μL. Thermal cycling conditions were 10 minutes at 95 °C, followed by 40 cycles of 95 °C for 15 second and 60 °C for 1 minute. A standard curve, ranging from 1 × 10^6^ to 5 copies per reaction of a diluted gBlock® (IDT®, Iowa, USA) containing targets for the IP2IP4 assay or diluted purified crAssphage amplicons (produced with DNA Clean and Concentrator^TM^ 25, ZYMO, USA) was used to convert Ct values to gene copies per reaction. Nucleic acid concentrations for gBlocks and purified amplicons were measured using a Qubit® 3.0 Fluorometer (Invitrogen®), allowing copy number to be determined from the known length of the amplicon or gBlock. Samples were quantified using either PCR plate specific standard curves or a composite standard curve from recent plates (Table 2). All qPCR reactions were carried out on either QuantStudio® 3 or QuantStudio® 5 (ThermoFisher®) real-time PCR systems.

**Table 2:** qPCR Assay Performance Parameters from a Composite of Eight Standard Curves

**Assay**	**R^2^**	**Intercept**	**Slope**	**Efficiency**	**LOQ**^a^**(copies/rxn)**
IP2IP4	0.97-0.99	39.228	-3.573	0.91	5
CPQ_056	0.99	40.162	-3.451	0.95	5

a LOQ determined as the lowest concentration at which ≥95% of reactions (out of 24) amplified successfully

### 2.6 Quality assurance

For all days on which wastewater samples were purified via ultracentrifugation, at least one processing blank was prepared by processing 20 mL distilled water instead of wastewater and measuring levels of SARS-CoV-2 RNA, crAssphage DNA, and crAssphage RNA. Throughout the study, 2 of 22 processing blanks contained quantifiable levels of crAssphage DNA (mean Ct = 36.019 ± 2.259) which was several orders of magnitude less than crAssphage quantities obtained from wastewater influent samples (mean Ct = 22.662 ± 1.418). For SARS-CoV-2, 2 of 22 processing blanks showed some degree of amplification, with one being quantifiable (June 10^th^, 2020, Ct = 35.637 ± 0.192, appx. 10 copies/mL). No processing blanks had amplification for crAssphage cDNA.

Following ultracentrifugation, at least one additional blank was prepared during total nucleic acid extraction by substituting 200 μL dissolved pellet with 200 μL molecular grade water. Throughout the study, 1 of 18 extraction blanks contained quantifiable levels of crAssphage DNA (Ct = 35.982 ± 0.545). For SARS-CoV-2, 1 of 18 extraction blanks contained quantifiable RNA (June 9^th^, 2020, Ct = 34.323 ± 0.515, appx. 24 copies/mL). No extraction blanks contained detectable levels of crAssphage cDNA. Due to suspected SARS-CoV-2 contamination, data from 9^th^ and 10^th^ of June 2020 were omitted from our analysis, effectively reducing the number of samples analyzed from 181 to 169.

For RT-qPCR and qPCR, plates contained at least three no template control (NTC) reactions. Throughout the study, 1 of 128 (0.8%) IP2IP4 NTC wells amplified (Ct = 40.957) and 9 of 198 (4.5%) of CPQ_056 NTC wells amplified. For CPQ_056, six of these NTC amplifications occurred on May 11^th^, 2020. On this plate, wastewater samples had a mean CPQ_056 Ct of 23.350 and positive NTCs had a mean Ct of 38.580 which suggests that contamination did not greatly affect estimates of crAssphage from wastewater on this run. Therefore, since the sole IP2IP4 NTC that showed amplification was >40 cycles and the few positive CPQ_056 NTCs were largely isolated to one plate and represented DNA quantities several orders of magnitude less than our wastewater nucleic acid extracts, no data were excluded from analysis based on the assessment of NTCs.

Kinetic outlier detection (KOD) was performed as described previously (Green and Field, 2012; Kirtane et al., 2019; Tichopad et al., 2010) on all 3,032 reactions to determine if qPCR inhibition affected the amplification of SARS-CoV-2 or crAssphage nucleic acid targets. Raw fluorescence data from each well were log-transformed and fit to a 4-parameter sigmoidal model using the *pcrbatch* function in R package *qpcR* version 1.4-1 (Ritz and Spiess, 2008; Spiess, 2018). We then estimated the first and second derivative maxima of each fitted model. Using a 10 Ct difference between the first and second derivative maxima as a quality criterion (i.e., “uni2” criteria in function *pcrbatch*), KOD analysis indicated that all wells with a Ct value < 45 (maximum possible Ct value) displayed no signs of inhibition. Inherent in these methods is the assumption that DNA polymerase and reverse transcriptase are equally susceptible to PCR inhibitors. Nonetheless, the total absence of signs of qPCR inhibition as indicated by sensitive KOD methods suggests that the purification methods used were effective at removing compounds that commonly affect qPCR amplification.

### 2.7 Integration of COVID-19 case data

COVID-19 testing data, consisting of all diagnostic tests including PCR and antigen-based methods, were obtained from The Electronic Clinical Laboratory Reporting System (ECLRS) (NYSDOH, 2020a,b). All test results were classified as positive, negative, inconclusive, or invalid. After excluding tests for out of state residents, all tests for COVID-19 during the study period were geocoded using the New York State Street Address Maintenance (SAM) Program (“NYS Street Address Mapping (SAM),” 2020). Additional geocoding was performed with geocoders from SAS and MapMarker to improve accuracy. Shape files of each service area were obtained from each corresponding municipality. Addresses occurring within the studied service areas were retained while addresses occurring outside the study area were excluded. Residences with private septics were identified using statewide tax parcel data from the New York State GIS Clearinghouse (“NYS GIS - Parcels,” 2020). Any addresses with private septics, which accounted for approximately 5% of COVID-19 tests, were excluded from the analysis. A daily count of positive test results by service area was tabulated after excluding inconclusive or invalid results. Human subject involvement with regards to COVID-19 diagnostic testing was approved by the New York State Department of Health's Institutional Review Board.

### 2.8 Data Interpretation and Analysis

To aid interpretation, SARS-CoV-2 wastewater RNA levels were classified into three distinct categories prior to data analysis. Samples that had all three qPCR replicates amplify above the LOQ of 5 genome copies per reaction were classified as quantifiable. Because both assays were able to amplify 5 copies per reaction consistently, samples that had at least one qPCR replicate amplify with a Ct < 40 were considered detected but not quantifiable (DNQ). Many of the samples classified as DNQ had one or two qPCR replicates above the LOQ of 5 copies but were still conservatively classified as DNQ for our analysis. Samples that had no amplification in any of the three wells (i.e., all three wells were “Undetermined”, Ct > 45) were considered below the limits of detection (BLOD; i.e., a “negative” sample).

To facilitate comparison of crAssphage concentrations between service areas, we used prior 24-hour flow and population serviced to calculate a per capita crAssphage nucleic acid load as follows:

$$\begin{array}{ccc} & & Per\;Capita\;Nucleic\;Acid\;Load\\ & & =\frac{Genome\;Copies\;per\;Liter\;X\;Daily\;Flow\;\left(L\right)\;}{Population\;Served\;\left(n\;persons\right)} \end{array}$$

Per capita nucleic acid load represents the estimated average daily contribution of crAssphage nucleic acids by an individual.

Pair-wise t-tests were used to test for significant differences in mean recovery using different sucrose concentrations and spin times. Conditional inference trees (CTrees) were developed using the partykit (version 1.2-10) package in R (version 1.2.5019) as done previously (Weller et al., 2020, WHO Coronavirus Disease (COVID-19) Dashboard [WWW Document] n.d, Wölfel et al., 2020) to assess the effects of service area size, average influent temperature, and pH on crAssphage DNA and RNA concentrations (R code available at https://github.com/Maxwell-Wilder/Co-quantification-of-crAssphage-increases-confidence-in-wastewater-based-epidemiology-for-SARS-CoV-2). Transit times were also used a predictor variable, but only for sites 601, 604, 605, 606, 617, and 619 as available (Wang et al., 2020).

## 3 Results

### 3.1 Optimizing recovery of viral nucleic acids

In an assessment of sucrose concentration and ultracentrifugation (“spin”) time, we found that a 50% cushion paired with a 90-minute spin time yielded greater crAssphage DNA concentrations than both a 20% cushion paired with a 20-minute spin time and a 70% cushion paired with a 150-minute spin time (p < 0.05, Table 3).

**Table 3:** Assessment of sucrose concentration and ultracentrifugation time on crAssphage DNA recovery.

**Sucrose Concentration**	**Spin Time (Minutes)**	**Replicate Tube**	**crAssphage DNA (Copies/L WW Source +/- SD)**
20 %	20	1	1.95 × 10^7^ (4.40 × 10^5^)
		2	2.53 × 10^7^ (5.95 × 10^5^)
50 %	90	1	9.04 × 10^7^ (3.56 × 10^6^)
		2	1.27 × 10^8^ (1.88 × 10^6^)
70 %	150	1	3.26 × 10^7^ (3.41 × 10^5^)
		2	7.52 × 10^7^ (8.55 × 10^5^)

We then assessed the impact of 30, 45, and 75-minute spin times on crAssphage nucleic acid recovery using a 50% sucrose cushion in an attempt to reduce processing time. We found that while both 45 and 75-minute spin times yielded greater quantities of crAssphage DNA than a 30-minute spin (p = <0.01, Table 4), there was no significant difference in crAssphage DNA recovery between 45 and 75-minute spins. Quantifiable amounts of crAssphage RNA were only recovered with a 45-minute spin time (Table 4). Low quantities of crAssphage RNA (DNQ) are potentially due to degradation, as RNA may have degraded while the wastewater sample was stored at 4 °C for approximately 5 days. Because 75-minute and 45-minute spin times yielded similar results, with 45-minutes being the only treatment to recover quantifiable crAssphage RNA, we proceeded with this spin time for further experiments and the analysis of wastewater samples.

**Table 4:** Assessment of ultracentrifugation time on crAssphage nucleic acid recovery using a 50% sucrose cushion.

**Spin Time (Minutes)**	**Replicate Tube**	**crAssphage DNA (Copies/L WW Source +/- SD)**	**crAssphage RNA (Copies/L WW Source +/- SD)**
30	1	6.74 × 10^7^ (1.57 × 10^6^)	DNQ
	2	7.21 × 10^7^ (4.47 × 10^6^)	BLOD
45	1	9.89 × 10^7^ (4.16 × 10^6^)	3.46 × 10^4^ (6.72 × 10^3^)
	2	9.90 × 10^7^ (2.82 × 10^6^)	DNQ
75*	1	9.39 × 10^7^ (5.86 × 10^6^)	DNQ
	2	1.19 × 10^8^ (3.80 × 10^6^)	DNQ

⁎ Among-treatment crAssphage DNA recovery was statistically different only for the 75-minute spin time (p = 0.005).

Having determined an optimal sucrose concentration and ultracentrifugation time, we then determined the approximate nucleic acid recovery for the total process using spiked heat deactivated SARS-CoV-2 (BEI Resources^®)^ and native crAssphage DNA and RNA as surrogates. Quantifiable levels of SARS-CoV-2 RNA and crAssphage RNA were found only in the pellet indicating that the majority of viral RNA is likely pelleted under these conditions (Table 5). Trace levels of RNA recovered from the cushion interface (SARS-CoV-2) and in the sucrose layers (crAssphage) suggest that low levels of SARS-COV-2 RNA also remain unpelleted. While quantifiable levels of crAssphage DNA were present in most layers post-ultracentrifugation, quantities found in the pellet were far greater than that of any other layer (p<0.001, Table 5). Although the magnitude of crAssphage DNA recovered from the pellet varied significantly among the two replicates (p = 0.002), SARS-CoV-2 RNA recovery was not statistically different. Based on the amount initially spiked, we estimated that 12% (s.d. = 5.5%) of deactivated SARS-CoV-2 RNA was recovered after both ultracentrifugation and nucleic acid extraction processes. A follow-up experiment in which BCoV RNA was added to pellets resulted in an average extraction recovery of 6.89% (s.d. = 1.58%) suggesting that the majority of nucleic acid loss in the total process occurred at the nucleic acid extraction step.

**Table 5:** Viral nucleic acids recovered from aqueous layers, cushion interface, cushion layers, and the pellet. Nucleic acid quantities are the total number of copies recovered from 200 uL sub samples of each layer.

**Layer**	**Volume (mL)**	**Replicate Tube**	**SARS-CoV-2 RNA (Copies +/- SD)**	**crAssphage RNA (Copies +/- SD)**	**crAssphage DNA (Copies +/- SD)**
Aqueous Upper	10	1	BLOD	BLOD	DNQ
		2	BLOD	BLOD	DNQ
Aqueous Lower	9	1	BLOD	BLOD	2.30 × 10^2^ (6.36 × 10^1^)
		2	BLOD	BLOD	DNQ
Cushion Interface	1.5	1	BLOD	BLOD	4.10 × 10^3^ (7.07 × 10^1^)
		2	DNQ	BLOD	8.59 × 10^3^ (3.97 × 10^2^)
Sucrose Upper	6	1	BLOD	DNQ	1.13 × 10^4^ (3.92 × 10^2^)
		2	BLOD	BLOD	4.72 × 10^3^ (5.43 × 10^3^)
Sucrose Lower	6	1	BLOD	DNQ	1.88 × 10^4^ (1.24 × 10^3^)
		2	BLOD	DNQ	8.19 × 10^3^ (4.71 × 10^2^)
Pellet	0.2	1	1.37 × 10^3^ (7.39 × 10^2^)	1.60 × 10^3^ (3.57 × 10^2^)	2.26 × 10^6^ (7.93 × 10^4^)
		2	1.42 ×10^3^ (7.19 ×10^2^)	DNQ	3.54 × 10^6^ (1.80 × 10^5^)

### 3.2 Abundance of SARS-CoV-2 and crAssphage in wastewater samples

While the vast majority of crAssphage DNA and RNA values fell within the quantifiable range, most SARS-CoV-2 RNA levels were either DNQ (49%) or BLOD (34%, Table 6). We were able to detect or quantify SARS-CoV-2 RNA from wastewater in 111 of the 169 samples that were analyzed over the study period. Of these 111 samples, 29 had quantifiable levels of SARS-CoV-2 RNA. In these samples, the average quantity of SARS-CoV-2 RNA recovered was 2.16×10^4^ (s.d. = 2.11 ×10^4^) genome copies per L of wastewater while the maximum observed quantity was 1.02 ×10^5^ (s.d. = 7.96 ×10^3^) genome copies per L of wastewater. crAssphage nucleic acids were quantifiable from the vast majority of wastewater samples (Table 6). Over the course of the study period, the average and maximum quantities of crAssphage DNA recovered were 2.05 × 10^8^ (s.d. = 2.18 ×10^8^) and 1.73 × 10^9^ (s.d. = 7.72 × 10^7^) genome copies per L of wastewater. For crAssphage RNA, the average and maximum quantities recovered were 4.00 × 10^5^ (s.d. = 4.61 × 10^5^) and 2.88 × 10^7^ (s.d. = 1.47 × 10^5^) genome copies per L.

**Table 6:** Percent of wastewater samples with quantifiable, detectable, and non-detectable amounts of nucleic acid target

**Target**	**Quantifiable (%)**	**DNQ (%)**	**BLOD (%)**
crAssphage DNA	100	0	0
crAssphage RNA	93	6	1
SARS-CoV-2 RNA	17	49	34

### 3.3 Association between crAssphage loads, influent flow, and population served

**Fig. 2:**
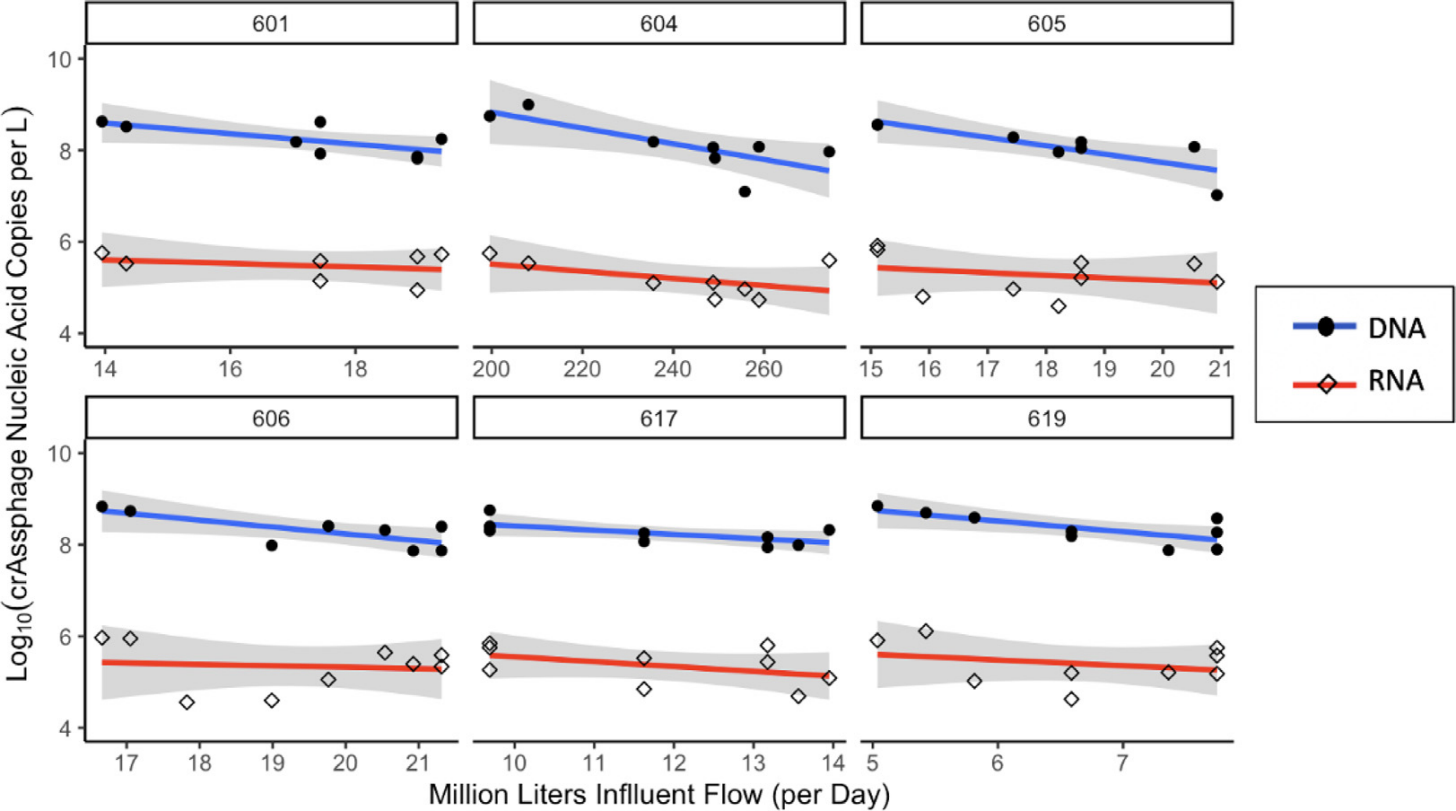
Relationship between crAssphage nucleic acid concentration (copies per L) and daily influent flow at six Onondaga County access points. crAssphage DNA concentration displays a significant negative relationship with influent flow (p < 0.05 each site except 617 where p = 0.052). crAssphage RNA concentration did not have a significant relationship with flows at any site.

**Fig. 3:**
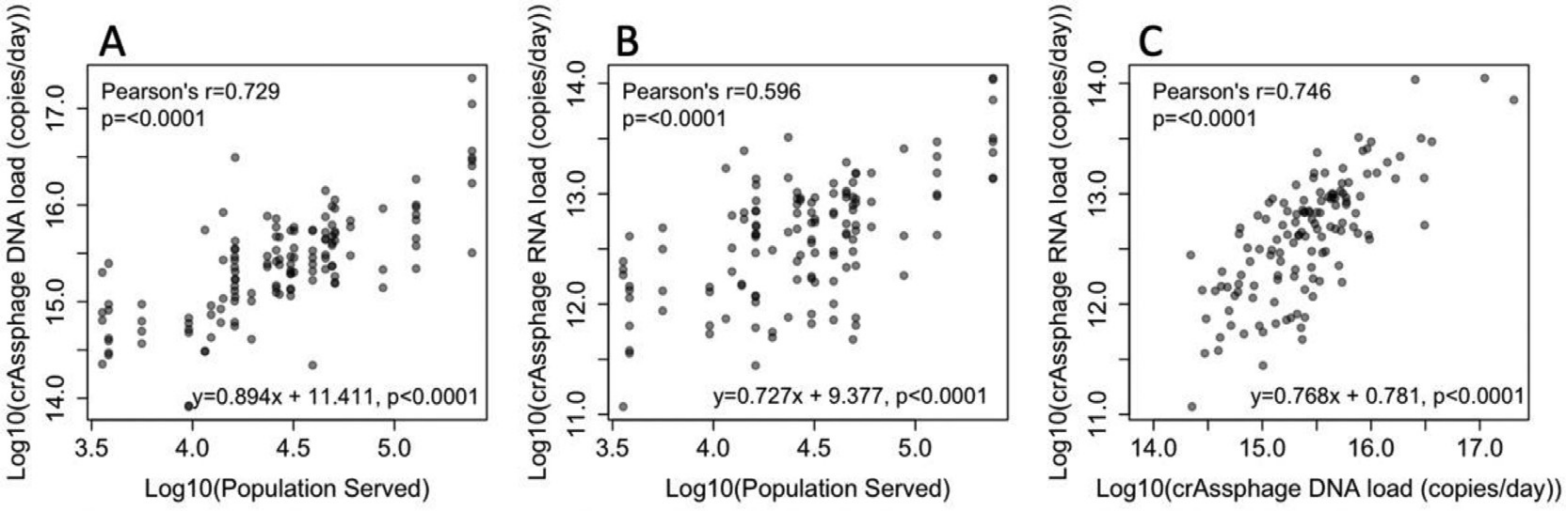
Relationship between crAssphage DNA load and population served (A), crAssphage RNA load and population served (B), and crAssphage RNA load and crAssphage DNA load (C). Load is the product of nucleic acid concentration and flow rate.

We observed a significant negative relationship between crAssphage concentrations detected and influent wastewater flow rates across all sites (Table S3). We selected six sites with the greatest number of sampling events (n=9 each) to look at this relationship on an individual basis and found significant negative relationships between crAssphage DNA concentration and flow, but no significant relationship between crAssphage RNA concentration and flow, potentially due to increased variability in crAssphage RNA measurements (Fig. 2, Table S3). Lower crAssphage concentrations during higher flow rates are likely attributable to wastewater dilution though sources such as groundwater infiltration and stormwater runoff, although the relative contribution of these sources in each service area is difficult to quantify. We also found significant relationships between crAssphage DNA and RNA loads and the population served in each service area (Fig. 3).

### 3.4 Variability in physical-chemical parameters and crAssphage loads between service areas

At each site (n = 8) where temperature and flow data were recorded, we observed a significant positive correlation between temperature and sampling date (Pearson's r = 0.94 – 0.97, p<0.05) and significant negative correlation between flow and sampling date (r = -0.82 – -0.96, p<0.05) reflecting the change toward warmer, dryer weather. At facilities 605 and 606, we also observed a significant negative correlation between pH and both sampling date (r = -0.75, -0.86, p<0.05) and water temperature (r = -0.76, -0.88, p<0.05), but at other sites no significant correlation was observed.

**Fig. 4:**
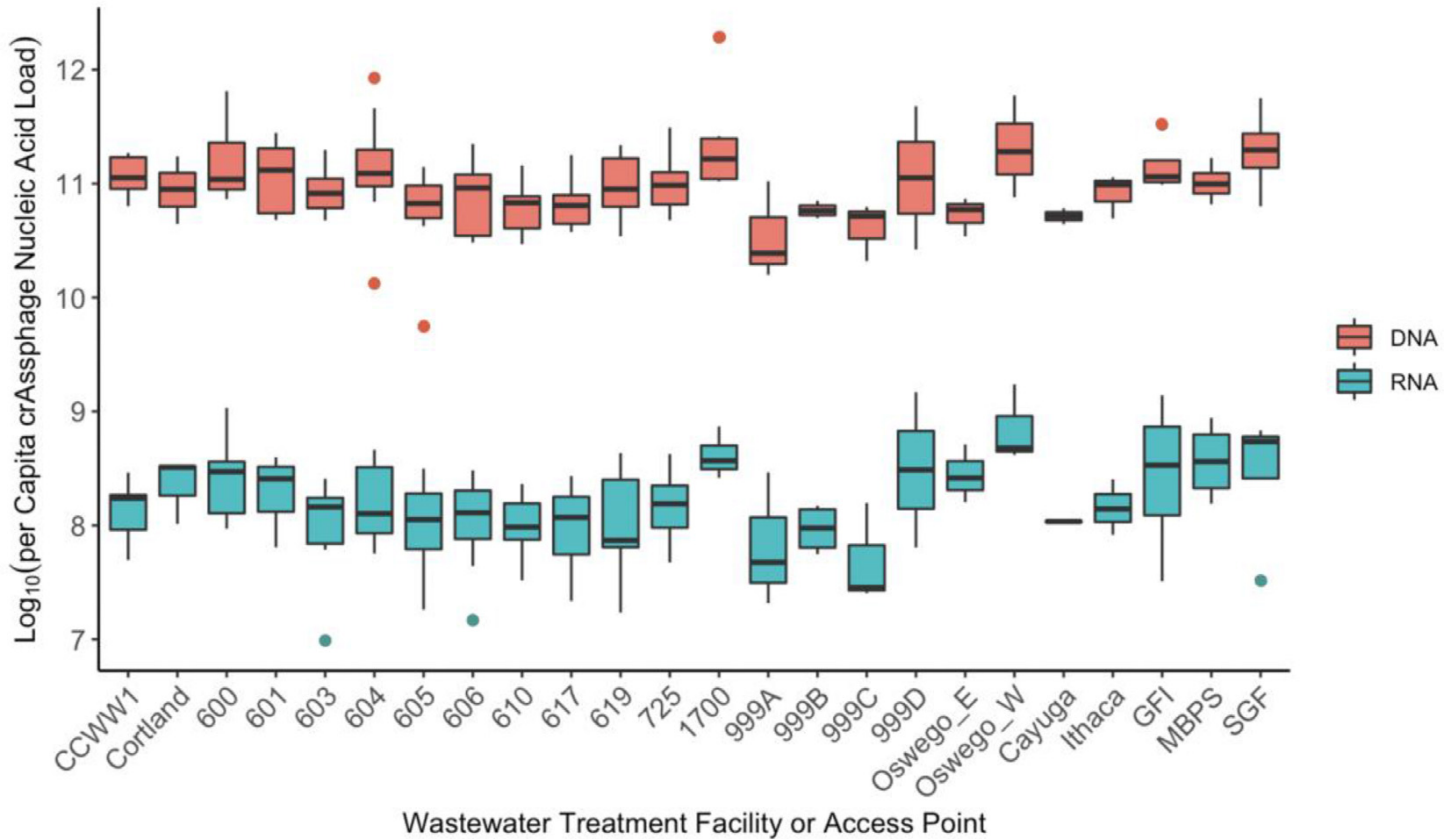
Variation in per capita crAssphage nucleic acid loads between sites.

On average, estimated per capita crAssphage contributions were 1.35 × 10^11^ genome copies per day (std. dev. = 1.99 × 10^11^) and 2.42 × 10^8^ genome copies per day (std. dev. = 2.77 × 10^8^) for DNA and RNA, respectively (Fig. 4). Based on regression analysis, we observed significantly lower per capita crAssphage DNA in service area 999A (4.85 × 10^10^, p=0.0365) compared to other sites. We also observed significantly higher crAssphage RNA per capita in service area Oswego_W (2.86 × 10^11^, p=0.0165) compared to other sites. Based on a pairwise comparison of service areas, no two sites had a significant difference in mean crAssphage DNA (p > 0.11; Tukey HSD, 95% CI) or mean crAssphage RNA (p > 0.09), although the sample size (Table S1) is too low at most sites to conclude no difference in means.

**Fig. 5:**
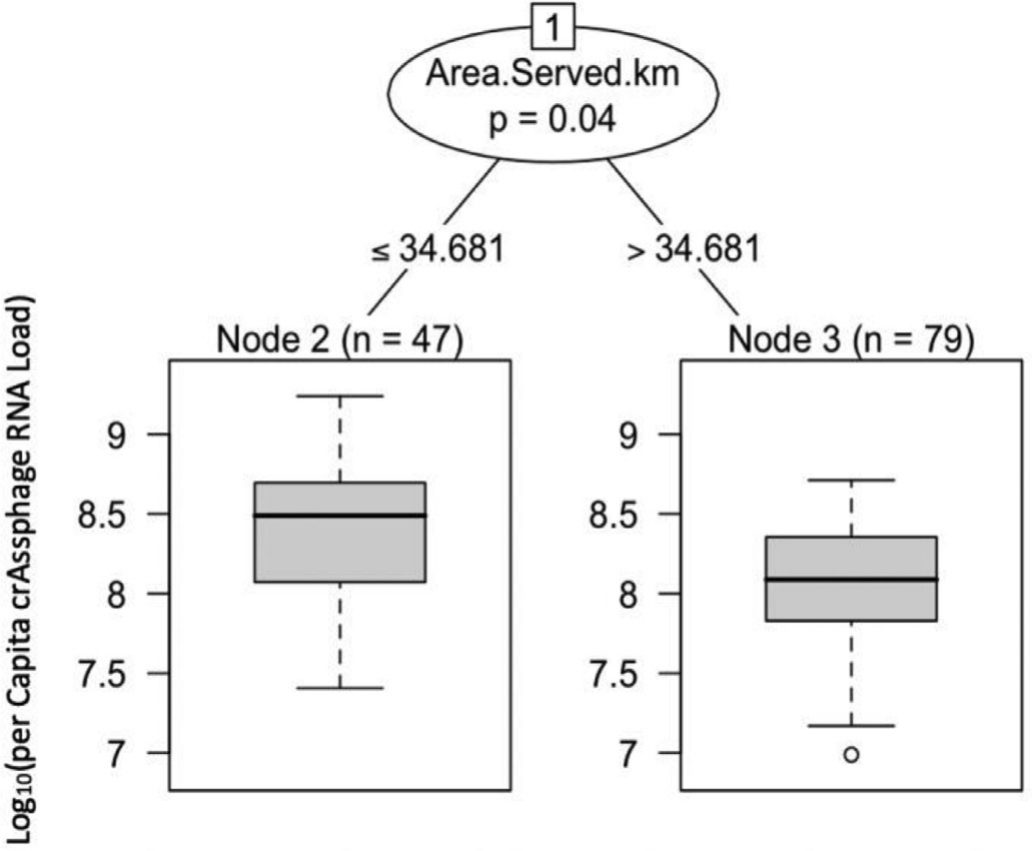
Association between smaller service areas and greater per capita crAssphage RNA load identified through conditional inference trees.

Using CTrees, we found that some of this variability in per capita crAssphage RNA loads could be explained by service area size. Per capita crAssphage RNA loads were 2.09 × 10^8^ gene copies higher in service areas smaller than 34.681 km^2^ (Fig. 5). A similar association was not observed for per capita DNA loads. No significant splits were identified when using average influent temperature, pH, or transit time as predictors for DNA or RNA loads as outcomes.

### 3.5 Association between SARS-CoV-2 concentrations and COVID-19 incidence following wastewater sample collection

Over all study areas, the highest positive test rates for which SARS-CoV-2 RNA remained BLOD corresponded to a weekly positive test rate of 12.4% and a weekly average of 2.19 daily positive tests per 10,000 population. The highest weekly positive test rates for samples classified as BLOD or DNQ were 20.9% or 3.97 daily positive tests per 10,000 population. Weekly positive test rates corresponding to quantifiable samples ranged from 1.68- 15.11% or 0.37-5.95 daily positive tests per 10,000 population depending on the site.

**Fig. 6:**
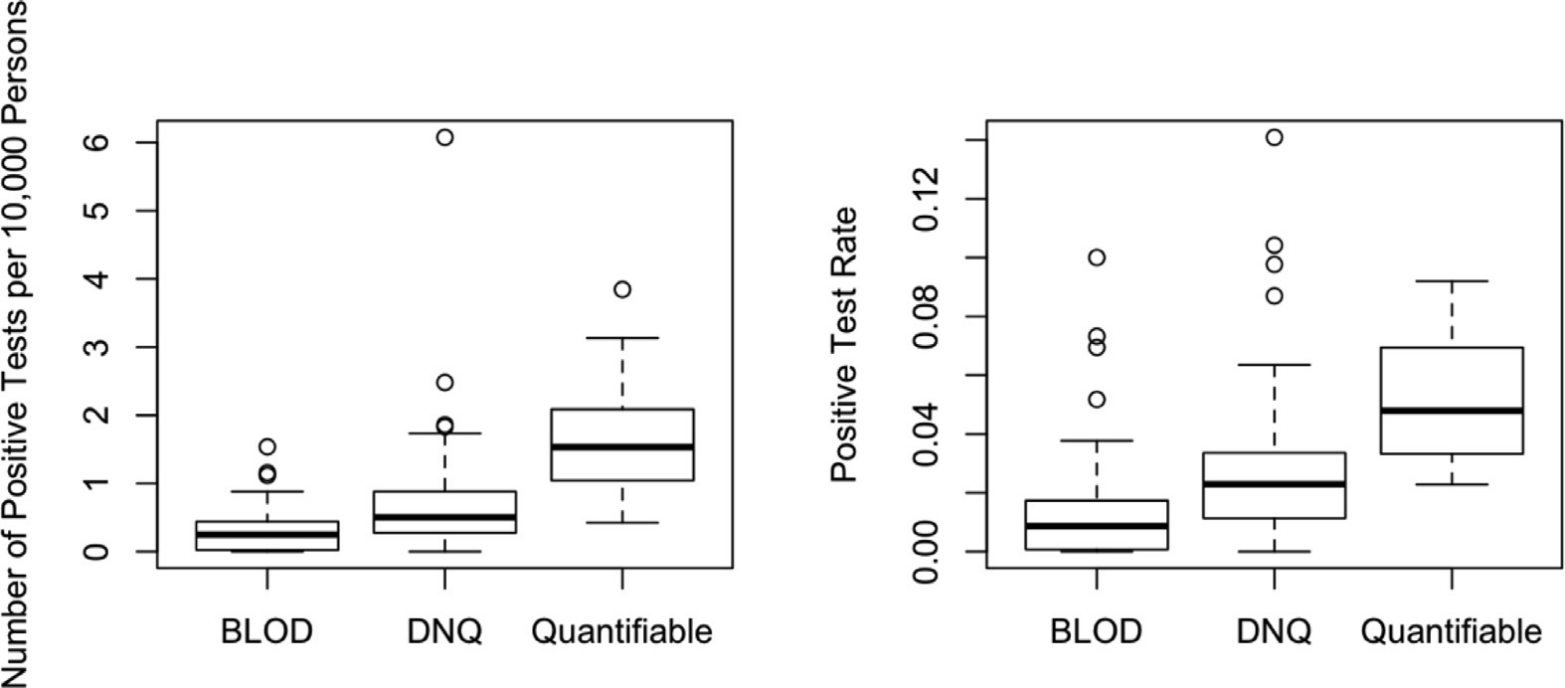
Association between SARS-CoV-2 RNA classification from wastewater and the average daily number individuals to test positive for COVID-19 (per 10,000 people) (left) and the testing positivity rate (right) among people contributing to a sewershed in the seven days following sample collection.

From a qualitative perspective, samples with quantifiable levels of SARS-CoV-2 RNA were associated with higher levels of positive test results the week following sampling (Fig. 6). Over the seven days following wastewater sample collection, both the average number of new positive tests per 10,000 persons and the testing positivity rate were significantly higher in quantifiable samples than in samples classified as BLOD or DNQ for SARS-CoV-2 (Welch two-sample t-test, p < 0.001). Samples classified as DNQ also had significantly higher rates and case counts than BLOD samples (Welch two-sample t-test, p ≤ 0.002).

Although the number of samples with quantifiable levels of SARS-CoV-2 RNA was limited (n=29), we did observe a significant relationship between the ratio of SARS-CoV-2 RNA to crAssphage DNA (p = 0.005, R^2^ = 0.27) and the number of positive tests per 10,000 population. This relationship was somewhat improved after excluding samples for which no crAssphage RNA was recovered (p = 0.004, R^2^ = 0.31). A similar association was found between the SARS-CoV-2:crAssphage DNA ratio and the number of positive tests per 10,000 population the week following sampling (p = 0.003, R^2^ = 0.30), which also improved slightly after excluding samples with no recoverable crAssphage RNA (p = 0.004, R^2^ = 0.33). Interestingly, significant positive associations between ratios and test rates were identified only when testing was expressed as a proportion of the population served and not as a proportion of total tests conducted (i.e., test positivity). No significant linear associations were identified between SARS-CoV-2 wastewater RNA concentrations and epidemiological parameters without first normalizing to crAssphage DNA.

## 4 Discussion

### 4.1 Advantages of ultracentrifugation through a sucrose cushion

We developed a sensitive, rapid, and scalable method for the detection and quantification of SARS-CoV-2 wastewater RNA based on direct ultracentrifugation though a sucrose cushion. Inspired by the early results of Wurtzer and colleagues (Wurtzer et al., 2020), we sought to further capitalize on the ability of ultracentrifugation to remove low-density contaminants that could potentially interfere with subsequent nucleic acid extraction and qPCR. Despite loss of RNA in the extraction process, we were able to quantify SARS-CoV-2 in areas with less than 1 positive test per 10,000 individuals. With this approach, it is possible to obtain wastewater testing results for both SARS-CoV-2 and crAssphage within 4.5 hours of a sample being received. The major limiting factor to this method is both ultracentrifuge availability and capacity, as the rotor used here can hold only six samples. However, the use of small volumes of wastewater (only 20 mL per sample) provides advantages in terms of transport, storage, and biosafety, although the use of larger volumes of wastewater may improve sensitivity. Other groups have since found the method to outperform other common concentration procedures for the analysis of wastewater from individual facilities (Colosi et al., 2020). This sensitivity, relatively quick turnaround time, and limited dependence on supply chain continuity may be an attractive option for groups considering wastewater surveillance.

Different concentration and nucleic acid extraction approaches should also be considered in an attempt to improve upon the 7-12% recovery that we estimated in this study. While approaches other than ultracentrifugation have reported higher recovery rates and variable cost and processing times (Table S4), Colosi and colleagues (2020) recently found sucrose cushion-based ultracentrifugation using a fixed-angle rotor, which accommodates tubes with twice the sample volume used in this study, and a NucleoSpin® extraction kit to outperform both electropositive filtration and PEG-precipitation methods. While the ultracentrifugation approach described by Colosi et al. (2020) demonstrated success, it is difficult to compare our methodologies without a direct measurement of nucleic acid percent recovery.

Results from optimization trials indicate that viral particles in wastewater exist in a mixture of states and a range of sedimentation properties that are likely to change from sample to sample. More sensitive methods, but also improved interpretation of wastewater surveillance data, would be facilitated by a better understanding of the state of both SARS-CoV-2 RNA and surrogate nucleic acids within wastewater and, specifically, what proportion are a) contained within viral particles, b) released and dissolved, or c) released and bound to other particles. While some studies have explored viral associations to various wastewater particles in terms of size and charge (da Silva et al., 2008; Hejkal et al., 1981), variability in particle association between different types of viruses (Chahal et al., 2016, Chen et al., 2020, Colosi et al., 2020, Crits-Christoph et al., 2021, da Silva et al., 2008) suggests that both SARS-CoV-2 and surrogate viral particle associations may require specific study with an additional focus on the state(s) of nucleic acids. If some DNA or RNA is bound, knowing the size and mass of the particles and how they vary over time and across locations would greatly improve the precision of methods based on size (e.g., ultrafiltration), charge (e.g., electropositive filtration), or mass (e.g., ultracentrifugation). Additionally, variability in wastewater particle composition between service areas and/or sampling locations may affect viral decay, as different particle associations have been shown to impact the survival of pathogens (as reviewed in Chahal et al. 2016). Bivins et al., (2020) estimated that 90% of SARS-CoV-2 RNA is degraded after 3.3 days in wastewater. A better understanding of how particle associations alter decay these rates is needed.

### 4.2 Epidemiologically relevant limits of detection

Although method performance varied across sites, SARS-CoV-2 wastewater RNA could be quantified in some areas experiencing as low as a 1.68% positivity rate. The method's ability to quantify such low levels of SARS-CoV-2 RNA suggests that the results are likely to be useful in managing public health responses at the initial stages of community spread, making this an important public health tool for COVID-19 surveillance. Our observation that SARS-CoV-2 RNA detection was associated with a higher incidence of COVID-19 in the next seven days further supports the use of wastewater surveillance as an early warning system with the amount of early warning dependent on a wide range of factors including frequency of wastewater sampling, site and sample characteristics affecting the sensitivity of detection, and the rate of the spread of infection. Further characterization of the service areas themselves and their wastewater infrastructure is needed to determine more precisely the areas where WBE for SARS-CoV-2 would be most useful.

### 4.3 crAssphage as a normalizer for spatiotemporal variability

Quantification of a surrogate organism in addition to SARS-CoV-2 can not only serve as a quality assurance measure, ensuring sufficient amounts of nucleic acids are recovered, but can also be used to normalize measured SARS-CoV-2 values to help account for the fluctuating concentrations of fecal material in wastewater. Our observation that SARS-CoV-2:crAssphage DNA ratios were significantly associated with the number of positive tests per 10,000 individuals, both 7 days before and after sampling, supports the use of crAssphage as a surrogate for SARS-CoV-2. As the significance of this association was improved following the exclusion of samples from which crAssphage RNA was not recovered, the quantification of both DNA and expressed RNA may be advantageous when using a DNA virus as a surrogate. Other viruses, such as pepper mild motile virus (PMMoV), have been used to facilitate the interpretation of SARS-CoV-2 WBE data (D'Aoust et al., 2021b, 2021a; Gerrity et al., 2021, Green et al., 2020, Green and Field, 2012; Jafferali et al., 2021; F. Wu et al., 2020b). Despite being an RNA virus like SARS-CoV-2, PMMoV is a rod-shaped virus that is very stable in the environment and has high temperature tolerance and resilience in adverse physiochemical conditions (Kitajima et al., 2018), which likely contributes to its relatively consistent abundance across wastewater treatment facilities (D'Aoust et al., 2021b, 2021a). In contrast, our results show that crAssphage nucleic acid concentrations are somewhat reflective of site differences and that crAssphage DNA concentrations respond to changes in flow and crAssphage RNA fluctuates in part as a function of sewershed area. Lower crAssphage RNA levels from larger sewersheds likely reflects the relative instability of RNA and suggests that measures of sewershed area, or other proxies for waste transit time, could help link SARS-CoV-2 wastewater RNA levels to relevant epidemiological parameters. More studies comparing crAssphage, PMMoV, and other surrogates are needed in order to determine which most accurately reflects the behavior of SARS-CoV-2 in wastewater. The assay we used to target crAssphage, CPQ_056, has been shown to cross-react with poultry litter (Ahmed et al., 2018). However, CPQ_056 marker concentrations were over 2-3 orders of magnitude lower in poultry litter compared to untreated wastewater and likely had little effect on our quantification of crAssphage nucleic acids. Nonetheless, use of this marker in areas heavily affected by poultry fecal contamination is not recommended.

### 4.4 Future work

In addition to the research needs relevant to particle binding and decay, as well as the continued methodological and processing refinements mentioned previously, SARS-CoV-2 strain identification from wastewater should be a priority of ongoing WBE for COVID-19. While sequencing approaches have been used successfully to detect variants from wastewater (Crits-Christoph et al., 2021; Jahn et al., 2021, Kirtane et al., 2019), these methods are relatively low throughput. The rapid development of standardized PCR-based assays to detect variants from wastewater would allow sensitive and early detection of variants that pose elevated health risks.

## Conclusions

• The ultracentrifugation-based method described here is a rapid and sensitive approach for the detection of SARS-CoV-2 in wastewater from areas with low numbers of COVID-19 cases.
• After normalization with crAssphage DNA, higher concentrations of SARS-CoV-2 wastewater RNA were significantly associated with positive COVID-19 tests the week following wastewater sample collection suggesting the approach could help predict near-term COVID-19 case levels.

## Funding

This work would not have been possible without seed funding from Syracuse University, the Environmental Data Science Initiative at SUNY-ESF, and the SUNY Discovery Fund. Additional funding was provided by Cooperative agreement EH171702 (Enhancing Innovation and Capabilities of the Environmental Public Health Tracking Network) through the Centers for Disease Control and Prevention.

## Declaration of Competing Interest

Provisional patent application no. 63/039,338 was filed on June 15, 2020 for which Quadrant Biosciences holds an exclusive license.

## Appendix Supplementary materials

Image, application 1

## References

[bib0001] Ahmed W., Angel N., Edson J., Bibby K., Bivins A., O'Brien J.W., Choi P.M., Kitajima M., Simpson S.L., Li J., Tscharke B., Verhagen R., Smith W.J.M., Zaugg J., Dierens L., Hugenholtz P., Thomas K.V., Mueller J.F. (2020). First confirmed detection of SARS-CoV-2 in untreated wastewater in Australia: A proof of concept for the wastewater surveillance of COVID-19 in the community. Science of The Total Environment.

[bib0002] Ahmed W., Bertsch P.M., Bivins A., Bibby K., Farkas K., Gathercole A., Haramoto E., Gyawali P., Korajkic A., McMinn B.R., Mueller J.F., Simpson S.L., Smith W.J.M., Symonds E.M., Thomas K.V., Verhagen R., Kitajima M. (2020). Comparison of virus concentration methods for the RT-qPCR-based recovery of murine hepatitis virus, a surrogate for SARS-CoV-2 from untreated wastewater. Sci Total Environ.

[bib0003] Ahmed W., Lobos A., Senkbeil J., Peraud J., Gallard J., Harwood V.J. (2018). Evaluation of the novel crAssphage marker for sewage pollution tracking in storm drain outfalls in Tampa. Florida. Water Research.

[bib0004] Kocamemi Alpaslan, B. Kurt, H. Hacioglu, S. Yarali, C. Saatci, A.M. Pakdemirli, B. (2020). First Data-Set on SARS-CoV-2 Detection for Istanbul Wastewaters in Turkey (preprint). Infectious Diseases (except HIV/AIDS).

[bib0005] Ampuero M., Valenzuela S., Valiente-Echeverria F., Soto-Rifo R., Barriga G.P., Chnaiderman J., Rojas C., Guajardo-Leiva S., Diez B., Gaggero A. (2020). SARS-CoV-2 Detection in Sewage in Santiago, Chile - Preliminary results. (preprint). Infectious Diseases (except HIV/AIDS).

[bib0006] Bar Or I., Yaniv K., Shagan M., Ozer E., Erster O., Mendelson E., Mannasse B., Shirazi R., Kramarsky-Winter E., Nir O., Abu-Ali H., Ronen Z., Rinott E., Lewis Y.E., Friedler E.F., Paitan Y., Bitkover E., Berchenko Y., Kushmaro A. (2020). Regressing SARS-CoV-2 sewage measurements onto COVID-19 burden in the population: a proof-of-concept for quantitative environmental surveillance (preprint). Epidemiology.

[bib0007] Barril P.A., Pianciola L.A., Mazzeo M., Ousset M.J., Jaureguiberry M.V., Alessandrello M., Sánchez G., Oteiza J.M. (2021). Evaluation of viral concentration methods for SARS-CoV-2 recovery from wastewaters. Science of The Total Environment.

[bib0008] Bivins A., Greaves J., Fischer R., Yinda K.C., Ahmed W., Kitajima M., Munster V.J., Bibby K. (2020). Persistence of SARS-CoV-2 in Water and Wastewater. Environ. Sci. Technol. Lett..

[bib0009] Chahal C., van den Akker B., Young F., Franco C., Blackbeard J., Monis P. (2016). Pathogen and Particle Associations in Wastewater: Significance and Implications for Treatment and Disinfection Processes. Adv Appl Microbiol.

[bib0010] Chen Y., Chen L., Deng Q., Zhang G., Wu K., Ni L., Yang Y., Liu B., Wang W., Wei C., Yang J., Ye G., Cheng Z. (2020). The presence of SARS-CoV-2 RNA in the feces of COVID-19 patients. Journal of Medical Virology.

[bib0011] Colosi, L.M., Barry, K.E., Kotay, S.M., Porter, M.D., Poulter, M.D., Ratliff, C., Simmons, W., Steinberg, L.I., Wilson, D.D., Morse, R., Zmick, P., Mathers, A.J., 2020. Development of wastewater pooled surveillance of SARS-CoV-2 from congregate living settings. medRxiv 2020.10.10.20210484. https://doi.org/10.1101/2020.10.10.20210484 10.1128/AEM.00433-21 PMC8316081 33858836

[bib0012] Crits-Christoph A., Kantor R.S., Olm M.R., Whitney O.N., Al-Shayeb B., Lou Y.C., Flamholz A., Kennedy L.C., Greenwald H., Hinkle A., Hetzel J., Spitzer S., Koble J., Tan A., Hyde F., Schroth G., Kuersten S., Banfield J.F., Nelson K.L. (2021). Genome Sequencing of Sewage Detects Regionally Prevalent SARS-CoV-2 Variants. mBio.

[bib0013] da Silva A.K., Le Guyader F.S., Le Saux J.-C., Pommepuy M., Montgomery M.A., Elimelech M. (2008). Norovirus removal and particle association in a waste stabilization pond. Environ Sci Technol.

[bib0014] D'Aoust P.M., Graber T.E., Mercier E., Montpetit D., Alexandrov I., Neault N., Baig A.T., Mayne J., Zhang X., Alain T., Servos M.R., Srikanthan N., MacKenzie M., Figeys D., Manuel D., Jüni P., MacKenzie A.E., Delatolla R. (2021). Catching a resurgence: Increase in SARS-CoV-2 viral RNA identified in wastewater 48 h before COVID-19 clinical tests and 96 h before hospitalizations. Science of The Total Environment.

[bib0015] D'Aoust P.M., Mercier E., Montpetit D., Jia J.-J., Alexandrov I., Neault N., Baig A.T., Mayne J., Zhang X., Alain T., Langlois M.-A., Servos M.R., MacKenzie M., Figeys D., MacKenzie A.E., Graber T.E., Delatolla R. (2021). Quantitative analysis of SARS-CoV-2 RNA from wastewater solids in communities with low COVID-19 incidence and prevalence. Water Research.

[bib0016] Daughton C. (2020). The international imperative to rapidly and inexpensively monitor community-wide Covid-19 infection status and trends. Science of The Total Environment.

[bib0017] Decaro N., Elia G., Campolo M., Desario C., Mari V., Radogna A., Colaianni M.L., Cirone F., Tempesta M., Buonavoglia C. (2008). Detection of bovine coronavirus using a TaqMan-based real-time RT-PCR assay. Journal of Virological Methods.

[bib0018] Farkas K., Hillary L.S., Malham S.K., McDonald J.E., Jones D.L. (2020). Wastewater and public health: the potential of wastewater surveillance for monitoring COVID-19. Current Opinion in Environmental Science & Health.

[bib0019] Gerrity D., Papp K., Stoker M., Sims A., Frehner W. (2021). Early-pandemic wastewater surveillance of SARS-CoV-2 in Southern Nevada: Methodology, occurrence, and incidence/prevalence considerations. Water Res X.

[bib0020] Green H., Wilder M., Middleton F.A., Collins M., Fenty A., Gentile K., Kmush B., Zeng T., Larsen D.A. (2020). Quantification of SARS-CoV-2 and cross-assembly phage (crAssphage) from wastewater to monitor coronavirus transmission within communities (preprint). Epidemiology.

[bib0021] Green H.C., Field K.G. (2012). Sensitive detection of sample interference in environmental qPCR. Water Res.

[bib0022] Hejkal T.W., Wellings F.M., Lewis A.L., LaRock P.A. (1981). Distribution of viruses associated with particles in waste water. Appl Environ Microbiol.

[bib0023] Jafferali M.H., Khatami K., Atasoy M., Birgersson M., Williams C., Cetecioglu Z. (2021). Benchmarking virus concentration methods for quantification of SARS-CoV-2 in raw wastewater. Science of The Total Environment.

[bib0024] Jahn K., Dreifuss D., Topolsky I., Kull A., Ganesanandamoorthy P., Fernandez-Cassi X., Bänziger C., Stachler E., Fuhrmann L., Jablonski K.P., Chen C., Aquino C., Stadler T., Ort C., Kohn T., Julian T.R., Beerenwinkel N. (2021). Detection of SARS-CoV-2 variants in Switzerland by genomic analysis of wastewater samples. medRxiv.

[bib0025] Kirtane A.A., Wilder M.L., Green H.C. (2019). Development and validation of rapid environmental DNA (eDNA) detection methods for bog turtle (Glyptemys muhlenbergii). PLOS ONE.

[bib0026] Kitajima M., Ahmed W., Bibby K., Carducci A., Gerba C.P., Hamilton K.A., Haramoto E., Rose J.B. (2020). SARS-CoV-2 in wastewater: State of the knowledge and research needs. Sci Total Environ.

[bib0027] Kitajima, M., Sassi, H.P., Torrey, J.R., 2018. Pepper mild mottle virus as a water quality indicator. npj Clean Water 1. https://doi.org/10.1038/s41545-018-0019-5

[bib0028] La Rosa G., Mancini P., Bonanno Ferraro G., Veneri C., Iaconelli M., Bonadonna L., Lucentini L., Suffredini E. (2020). SARS-CoV-2 has been circulating in northern Italy since December 2019: evidence from environmental monitoring (preprint). Infectious Diseases (except HIV/AIDS).

[bib0029] Larsen D., Dinero R.E., Asiago-Reddy E., Green H., Lane S., Shaw A., Zeng T., Kmush B. (2020). A review of infectious disease surveillance to inform public health action against the novel coronavirus SARS-CoV-2 (preprint). SocArXiv.

[bib0030] Mallapaty S. (2020). How sewage could reveal true scale of coronavirus outbreak. Nature.

[bib0031] McMinn B.R., Korajkic A., Kelleher J., Herrmann M.P., Pemberton A.C., Ahmed W., Villegas E.N., Oshima K. (2021). Development of a Large Volume Concentration Method for Recovery of Coronavirus from Wastewater. Science of The Total Environment.

[bib0032] Medema G., Heijnen L., Elsinga G., Italiaander R., Brouwer A. (2020). Presence of SARS-Coronavirus-2 in sewage (preprint). Occupational and Environmental Health.

[bib0033] Meselson, M., 2020. Droplets and Aerosols in the Transmission of SARS-CoV-2. https://doi.org/10.1056/NEJMc2009324 10.1056/NEJMc2009324 PMC7179963 32294374

[bib0034] Nemudryi A., Nemudraia A., Surya K., Wiegand T., Buyukyoruk M., Wilkinson R., Wiedenheft B. (2020). Temporal detection and phylogenetic assessment of SARS-CoV-2 in municipal wastewater (preprint). Epidemiology.

[bib0035] Nemudryi A., Nemudraia A., Wiegand T., Surya K., Buyukyoruk M., Cicha C., Vanderwood K.K., Wilkinson R., Wiedenheft B. (2020). Temporal Detection and Phylogenetic Assessment of SARS-CoV-2 in Municipal Wastewater. Cell Reports Medicine.

[bib0036] NYS GIS - Parcels [WWW Document], n.d. URL http://gis.ny.gov/parcels/(accessed 12.1.20).

[bib0037] NYS Street Address Mapping (SAM) [WWW Document], n.d. URL https://gis.ny.gov/streets/(accessed 12.1.20).

[bib0038] Polo D., Quintela-Baluja M., Corbishley A., Jones D.L., Singer A.C., Graham D.W., Romalde J.L. (2020). Making waves: Wastewater-based epidemiology for COVID-19 – approaches and challenges for surveillance and prediction. Water Research.

[bib0039] Randazzo W., Truchado P., Cuevas-Ferrando E., Simón P., Allende A., Sánchez G. (2020). SARS-CoV-2 RNA in wastewater anticipated COVID-19 occurrence in a low prevalence area. Water Research.

[bib0040] Rico M., Andrés-Costa M.J., Picó Y. (2017). Estimating population size in wastewater-based epidemiology. Valencia metropolitan area as a case study. Journal of Hazardous Materials, Special Issue on Emerging Contaminants in engineered and natural environment.

[bib0041] Ritz C., Spiess A.-N. (2008). qpcR: an R package for sigmoidal model selection in quantitative real-time polymerase chain reaction analysis. Bioinformatics.

[bib0042] Rusiñol M., Martínez-Puchol S., Forés E., Itarte M., Girones R., Bofill-Mas S. (2020). Concentration methods for the quantification of coronavirus and other potentially pandemic enveloped virus from wastewater. Curr Opin Environ Sci Health.

[bib0043] Singer, A., Wray, R., 2020. Detection and Survival of SARS-coronavirus in Human Stool, Urine, Wastewater and Sludge. https://doi.org/10.20944/preprints202006.0216.v2

[bib0044] Spiess, A.-N., 2018. qpcR: Modelling and Analysis of Real-Time PCR Data.

[bib0045] Stachler E., Kelty C., Sivaganesan M., Li X., Bibby K., Shanks O.C. (2017). Quantitative CrAssphage PCR Assays for Human Fecal Pollution Measurement. Environ. Sci. Technol..

[bib0046] Tichopad A., Bar T., Pecen L., Kitchen R.R., Kubista M., Pfaffl M.W. (2010). Quality control for quantitative PCR based on amplification compatibility test. Methods.

[bib0047] Vallejo, J.A., Rumbo-Feal, S., Conde-Perez, K., Lopez-Oriona, A., Tarrio, J., Reif, R., Ladra, S., Rodino-Janeiro, B.K., Nasser, M., Cid, A., Veiga, M.C., Acevedo, A., Lamora, C., Bou, G., Cao, R., Poza, M., 2020. Highly predictive regression model of active cases of COVID-19 in a population by screening wastewater viral load. medRxiv 2020.07.02.20144865. https://doi.org/10.1101/2020.07.02.20144865

[bib0048] Wang S., Green H.C., Wilder M.L., Du Q., Kmush B.L., Collins M.B., Larsen D.A., Zeng T. (2020). High-throughput wastewater analysis for substance use assessment in central New York during the COVID-19 pandemic. Environ. Sci.: Processes Impacts.

[bib0049] Weller D., Belias A., Green H., Roof S., Wiedmann M. (2020). Landscape, Water Quality, and Weather Factors Associated With an Increased Likelihood of Foodborne Pathogen Contamination of New York Streams Used to Source Water for Produce Production. Front. Sustain. Food Syst..

[bib0050] WHO Coronavirus Disease (COVID-19) Dashboard [WWW Document], n.d. URL https://covid19.who.int (accessed 10.23.20).

[bib0051] Wölfel R., Corman V.M., Guggemos W., Seilmaier M., Zange S., Müller M.A., Niemeyer D., Jones T.C., Vollmar P., Rothe C., Hoelscher M., Bleicker T., Brünink S., Schneider J., Ehmann R., Zwirglmaier K., Drosten C., Wendtner C. (2020). Virological assessment of hospitalized patients with COVID-2019. Nature.

[bib0052] Wu F., Xiao A., Zhang J., Moniz K., Endo N., Armas F., Bonneau R., Brown M.A., Bushman M., Chai P.R., Duvallet C., Erickson T.B., Foppe K., Ghaeli N., Gu X., Hanage W.P., Huang K.H., Lee W.L., Matus M., McElroy K.A., Nagler J., Rhode S.F., Santillana M., Tucker J.A., Wuertz S., Zhao S., Thompson J., Alm E.J. (2020). SARS-CoV-2 titers in wastewater foreshadow dynamics and clinical presentation of new COVID-19 cases (preprint). Infectious Diseases (except HIV/AIDS).

[bib0053] Wu, F., Zhang, J., Xiao, A., Gu, X., Lee, W.L., Armas, F., Kauffman, K., Hanage, W., Matus, M., Ghaeli, N., Endo, N., Duvallet, C., Poyet, M., Moniz, K., Washburne, A.D., Erickson, T.B., Chai, P.R., Thompson, J., Alm, E.J., 2020b. SARS-CoV-2 Titers in Wastewater Are Higher than Expected from Clinically Confirmed Cases 5, 9. 10.1128/mSystems.00614-20 PMC7566278 32694130

[bib0054] Wu Y., Guo C., Tang L., Hong Z., Zhou J., Dong X., Yin H., Xiao Q., Tang Y., Qu X., Kuang L., Fang X., Mishra N., Lu J., Shan H., Jiang G., Huang X. (2020). Prolonged presence of SARS-CoV-2 viral RNA in faecal samples. The Lancet Gastroenterology & Hepatology.

[bib0055] Wurtzer S., Marechal V., Mouchel J.-M., Maday Y., Teyssou R., Richard E., Almayrac J.L., Moulin L. (2020). Evaluation of lockdown impact on SARS-CoV-2 dynamics through viral genome quantification in Paris wastewaters (preprint). Epidemiology.

[bib0056] Zhang, D., Ling, H., Huang, X., Li, J., Li, W., Yi, C., Jiang, Y., He, Y., Deng, S., Zhang, X., Liu, Y., Li, G., Qu, J., 2020. Potential spreading risks and disinfection challenges of medical wastewater by the presence of Severe Acute Respiratory Syndrome Coronavirus 2 (SARS-CoV-2) viral RNA in septic tanks of fangcang hospital 17. 10.1016/j.scitotenv.2020.140445 PMC7308756 32599407

[2021] 2021. NOAA. (Accessed 15 February 2021).

[bib0058] 2020a. NYS DOH. (Accessed 1 December 2020).

[bib0059] 2020b. NYS DOH. (Accessed 1 December 2020).

